# First-principles study of the electrochemical properties of NaFeCl_4_ for cathode applications in sodium-ion batteries

**DOI:** 10.1039/d5ra06124e

**Published:** 2025-12-19

**Authors:** Suk-Gyong Hwang, Tae-Il Ri, Ryo-Gyong Choe, Chung-Hyok Rim, Chol-Jun Yu

**Affiliations:** a Computational Materials Design, Faculty of Materials Science, Kim Il Sung University Taesong District Pyongyang Democratic People's Republic of Korea cj.yu@ryongnamsan.edu.kp

## Abstract

All-solid-state sodium-ion batteries (ASSIBs) have attracted significant attention in the energy storage technology field, but the selection of an appropriate cathode material is indispensable. In this work, we perform first-principles calculations on the structural, electrochemical, electronic and lattice vibrational properties of NaFeCl_4_ for cathode applications in ASSIBs. Considering the crystalline symmetry, we perform bond valence sum analysis to predict additional positions for inserting Na atoms into a doubled supercell and determine the optimized structure with the lowest energy among different configurations for each Na composition. Our calculations demonstrate that the lattice constants of the stable intermediate phases Na_1+*x*_FeCl_4_ (*x* = 0–1) vary smoothly with increasing content of inserted Na *x*, confirming relative volume changes of less than 5%. We calculate the formation enthalpies of intermediate phases Na_1+*x*_FeCl_4_, plot the convex hull, and determine the step-like electrode potential, finding an average voltage of 3.45 V and a maximum specific capacity of 121 mAh g^−1^. Furthermore, we compute activation energies for Na-ion migrations along three different pathways and demonstrate fast Na-ion diffusion with low activation energy. Finally, the electronic band structures and phonon dispersions are investigated to estimate the electron conduction properties and thermodynamic stability. Our work paves the way for designing advanced cathode materials for ASSIBs using iron-based chloride materials.

## Abstract 2

We studied the structural, electronic, and lattice vibrational properties, as well as the ionic conductivity and electrode potential, of NaFeCl_4_ using first-principles calculations for cathode applications in all-solid-state sodium-ion batteries.

## Graphical abstract

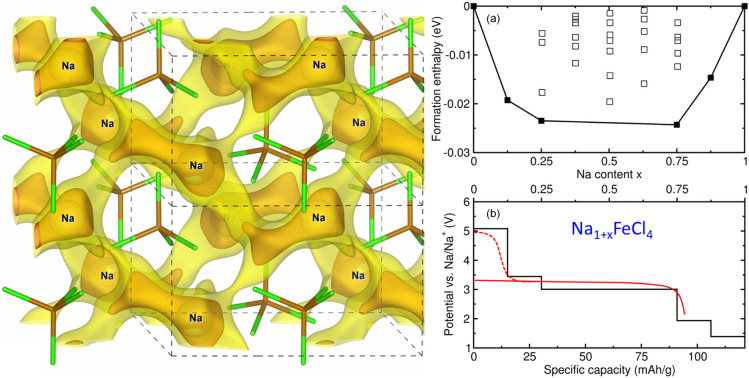

## 1 Introduction

Due to the increasing demands for clean energy and electric vehicles (EVs), advanced batteries with high-performance and low-cost have been attracting significant attention over the past decade.^1–3^ Lithium-ion batteries (LIBs) dominate the battery market at present, being widely used for EVs, as well as portable electronic devices, but their sustainable development is hindered by resource depletion, environmental damage and serious safety issues.^4–6^ In this context, sodium-ion batteries (SIBs) have been drawing a renewed interest owing to a high abundance of Na resources, low cost of the batteries, improved safety and the same operational mechanism as LIBs.^7–10^ In particular, all-solid-state sodium-ion batteries (ASSIBs) using various solid-state electrolytes (SSEs) have emerged as next-generation batteries with greatly increased energy density, much faster charging times and improved safety.^11,12^ However, new electrode materials, especially cathode materials with a good mechanical compatibility with SSEs and wide electrochemical stability window, are indispensable for commercially viable ASSIBs.^13–17^

While the liquid electrolytes in conventional batteries are often composed of flammable and hazardous solvents, SSEs consist of non-flammable polymers or ceramics, thereby eliminating the hazards of fire and explosion.^18^ In addition to enhanced safety, SSEs have been shown to inhibit dendrite penetration, making it possible to use Li or Na metal as the anode material and thus increase the gravimetric/volumetric energy densities. Although solid polymers have merits such as being soft and easy to process, low cost and good compatibility with electrodes, the ionic conductivity at room temperature (RT) is very low, requiring a high operating temperature of ∼80 °C.^19^ In this context, various inorganic solid materials in either a crystalline or glassy phase have been developed as potential SSEs for ASSIBs with their own advantages and disadvantages, such as oxides,^20,21^ sulfides,^22,23^ borohydrides,^24,25^ and halides.^26–35^

Among SSEs, chlorides in particular are considered the most promising candidates for enabling ASSIB technology due to several intrinsic chemical properties of the Cl^−^ anion.^27–30^ Compared with divalent anionic compounds such as layered oxides, sulfides and polyanion compounds, the monovalent halogen anion exhibits a weaker coulombic interaction with alkali cations during their migration, leading to faster Na-ion migration and thus higher ionic conductivity.^13^ Moreover, the ionic radius of the Cl^−^ anion (1.67 Å) is relatively larger than that of the O^2−^ anion (1.26 Å), implying that the chlorides have longer ionic bond lengths than the oxides, from which higher ionic mobility and higher deformability are expected.^14^ The higher deformability is especially beneficial to the mechanical compatibility with electrode materials and fabrication of ASSIBs as it allows a sinterless compaction process.^22^ Owing to the higher electrochemical redox potential, the chloride anion in particular can offer higher oxidative stability.^36^ This is connected with the higher oxidation resistance, which leads to the high working voltage required for the high energy density of batteries when used as a cathode material.^35,37^ However, the chlorides have a high polarity, causing dissolution of the constituent elements and thus lowering the cycle life. When the chloride electrodes are used in contact with solid electrolytes, the dissolution reaction is effectively suppressed, indicating that the chloride electrodes can be used as high-voltage cathode materials of ASSIBs. When compared with fluorides, a NaFeCl_4_ cathode has a higher potential (3.45 V *vs.* Na/Na^+^) than a Na_2_FeF_4_ cathode (3.0 V *vs.* Na/Na^+^), leading to a higher specific capacity (121 mAh g^−1^*vs.* 90 mAh g^−1^).^14,15^ Tanibata *et al.*^14^ synthesized the iron-based chloride NaFeCl_4_ for use as a cathode material in an ASSIB, which adopted the solid electrolyte (Na_3_PS_4_| Na_2.25_Y_0.25_Zr_0.75_Cl_6_) and a Na–Sn alloy anode.

In this work, we performed first-principles calculations on NaFeCl_4_ (NFC) as a cathode material for SIBs, aiming to gain atomistic insights into its electrochemical properties. Using the bond-valence sum (BVS) analysis, we identified possible positions for further Na-ion insertion into NFC and the pathways for Na-ion migration. Based on the identified initial and final phases of NFC during charge/discharge process, we evaluated the formation energies of the intermediate phases and plotted the convex hull curve, from which the theoretical electrode potential step was estimated as a function of specific capacity. The activation energies for Na-ion migration along the identified possible pathways were computed to reveal the mechanism of the charge/discharge process and to evaluate the ionic conductivity. We also performed electronic structure calculations to elucidate the electronic transport properties and phonon calculations to estimate the thermodynamic stability.

## 2 Methods

### 2.1 Materials modeling

**Fig. 1:**
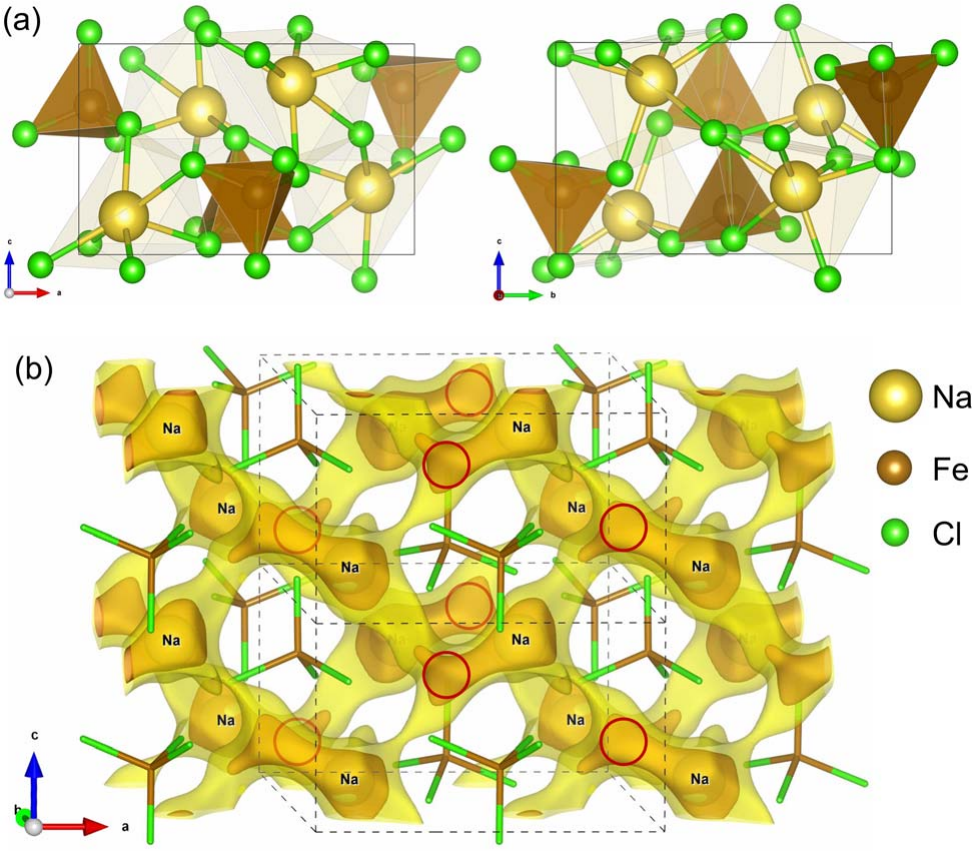
(a) Polyhedral view of the optimized unit cell and (b) isosurface view of the bond valence sum profile, where brown- and yellow-coloured isosurfaces correspond to *B*(***r***) = 2 and *B*(***r***) = 3, respectively, the red-coloured circles denote the provisional positions for inserting Na atoms, and dashed lines indicate the unit cell.

It is known that NFC crystallizes in an orthorhombic crystal structure with the space group *P*2_1_2_1_2_1_, which is registered in the ICSD (Inorganic Crystalline Solid Database) as entry #16994.^38^ Its crystalline structure is characterized by an isolated FeCl_4_ tetrahedron and 6-coordinated Na atoms with Na–Cl bond lengths varying from 2.79 to 3.08 Å, as shown in Fig. 1(a). The unit cell contains four formula units (fu) including 24 atoms and its experimental lattice constants are *a* = 10.304, *b* = 9.880 and *c* = 6.235 Å.^38^ To simulate the charge/discharge process by inserting/removing Na ions, the supercell was constructed by doubling the unit cell along the crystallographic *c* direction, resulting in 8 fu (48 atoms).

Iron-based compounds can act as electrode materials for rechargeable alkali-ion batteries based on the Fe^2+/3+^ redox reaction.^39–42^ In NFC, all Fe cations have a +3 oxidation state; therefore, additional Na^+^ cations can be inserted into the host so that the Fe^3+^ cations are reduced to the +2 oxidation state, corresponding to the discharge process of the cathode material in SIBs. Since the supercell containing eight fu is used for the simulation, there are an additional eight positions for inserting Na atoms. To identify the provisional positions for the additional Na atoms, we applied the bond valence sum (BVS) approach using the following equation:^43^1
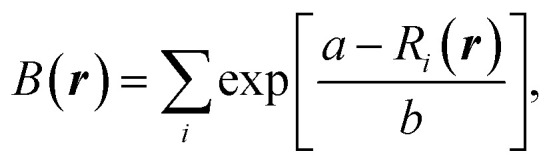
where *R*_*i*_(***r***) = |***r*** − ***R***_*i*_| is the position vector of the *i*-th atom *R*_*i*_ for the Na–Cl bond, *a* = 1.803 and *b* = 0.37.^44^Fig. 1(b) shows the isosurface plot of BVS in the unit cell. In this figure, the brown-coloured isosurface with *B*(***r***) = 2 resembles a slightly asymmetric dumbbell, whose larger end site is already occupied by a host Na atom, while the other end site will be used for inserting Na atom. For the supercell, *i.e.*, the doubled unit cell, we found eight dumbbells and thus eight positions for inserting Na atoms, denoted by red-coloured circles in Fig. 1(b). Therefore, the initial compound is the host NaFeCl_4_ where iron is in the Fe^3+^ oxidation state. After the discharge process, the final compound should be Na_2_FeCl_4_ with iron reduced to the Fe^2+^ oxidation state. The intermediate phases are denoted as Na_1+*x*_FeCl_4_ with *x* = 0–1. For this process, the theoretical specific capacity is determined to be *S* = *xeN*_A_/(*M*·*c*) = 121.25 mAh g^−1^, where *x* = 1 is the number of inserted Na atoms per fu, *M* = 220.649 g mol^−1^ is the molar mass of NaFeCl_4_, *e* is the elementary charge (1.6 × 10^−19^ C), *N*_A_ is Avogadro’s number (6.023 × 10^23^ mol^−1^), and *c* = 3.6 mAh C^−1^ is a constant. Furthermore, as shown in Fig. 1(b), the yellow-coloured isosurface corresponds to the larger BVS value of *B*(***r***) = 3, demonstrating the pathways for Na-ion migration.

### 2.2 Computational details

In this work, all the calculations were carried out by applying the pseudopotential plane-wave method within the density functional theory (DFT) framework, as implemented in the Quantum ESPRESSO (QE, version 7.2) package.^45,46^ To describe the coulombic ion-electron interactions, the ultrasoft pseudopotentials of all the atoms were used as provided from the GBRV library,^47,48^ with outer-shell electron configurations of Na-2s^2^2p^6^3s^1^, Fe-3s^2^3p^6^4s^2^3d^6^, Cl-3s^2^3p^5^. We applied the DFT+*U* method to consider the localized Fe 3d states with the effective Hubbard parameter *U*_eff_ = 2.0 eV.^49^ For the exchange–correlation (XC) interactions between the valence electrons, we used the optB88 functional, which consists of the Slater (*α* = 2/3) exchange, Perdew–Wang correlation,^50^ gradient exchange correction by Ortiz and Ballone^51^ and gradient correction on correlation by Perdew.^52^ The kinetic cutoff energies were set to 50 and 400 Ry for the wave function and electronic density, respectively. The *k*-point meshes for the Brillouin zone (BZ) integration were set to (2 × 2 × 4) and (2 × 2 × 2) for the unit cell and supercell, respectively. These computational parameters guarantee a total energy convergence of 5 meV per atom (see Fig. S1, SI). For the electronic structure calculations, a denser *k*-point mesh of (4 × 4 × 6) was used, while considering the spin-polarization effect with an antiferromagnetic configuration. Structural optimizations were performed with convergence thresholds for the forces on the atoms of 5 × 10^−4^ Ry per Bohr and a pressure on the lattice of 0.05 kbar, while not considering spin effects.

The phonon dispersion curves and phonon density of states (DOS) were calculated using the finite displacement method and the supercell, as implemented in the ALAMODE code^53^ in connection with the QE package. The anharmonic phonon effect was considered by performing calculations at finite temperatures ranging from 0 to 300 K with an interval of 100 K within the self-consistent phonon (SCP) theory.^54^ We used a *k*-point mesh of (4 × 4 × 4) and a *q*-point mesh of (10 × 10 × 10). The activation energies for Na-ion diffusion along the pathways predicted by BVS analysis were calculated using the climbing-image nudged elastic band (CI-NEB) method, as implemented in the QE package.^55^ Seven NEB images were employed based on the migration paths. During the NEB calculations, the size of the supercell was fixed at the optimized value, while all atoms were relaxed until the forces converged to 0.01 eV Å^−1^. The crystalline structures and BVS analysis were visualized using the VESTA code.^56^

## 3 Results and discussion

### 3.1 Structural properties

Firstly, we determined the lattice constants of the NFC unit cell with 4 fu (24 atoms) by using the different XC functionals with or without the *U* parameter (Table S1, SI). It was found that the optB88 XC functional with *U* = 2 eV produced the most reliable lattice constants, such as *a* = 10.184, *b* = 9.787 and *c* = 6.158 Å with relative errors of less than ±1.24% compared with the experimental values^38^ (see Table S2 for atomic coordinates, SI). With the optB88 XC functional and *U* = 2 eV, the mass density was determined to be 2.39 g cm^−3^, in good agreement with the experimental value of 2.31 g cm^−3^.^14,38^

**Fig. 2:**
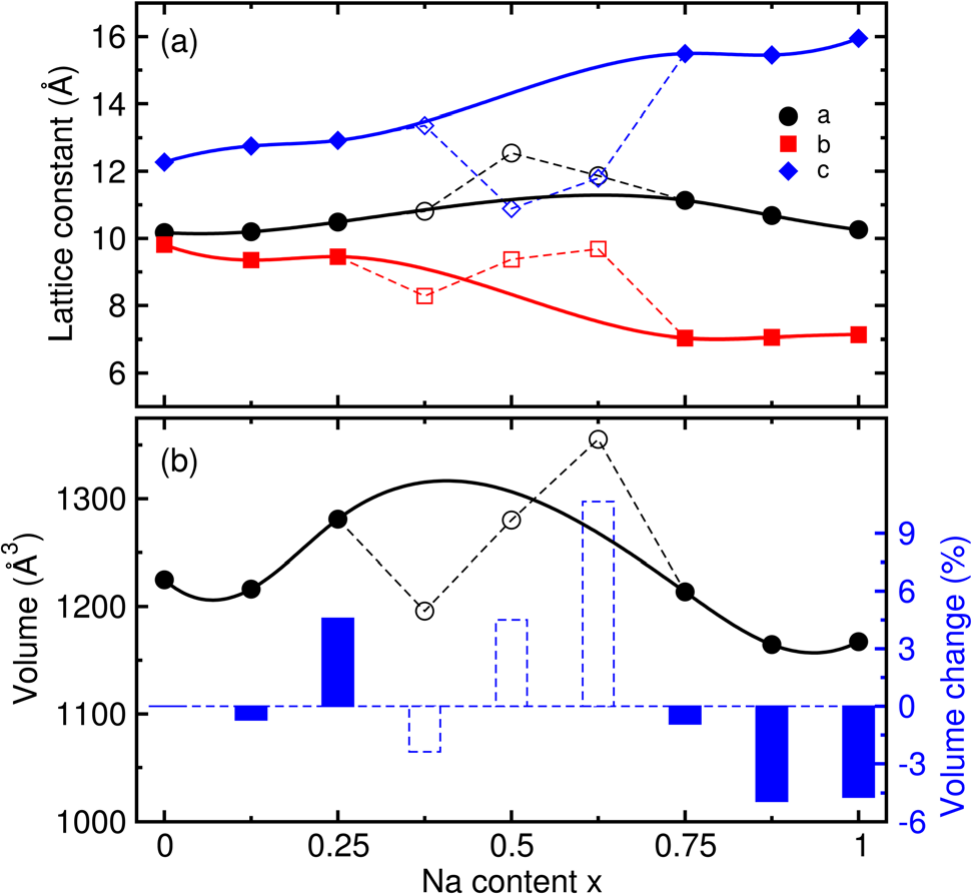
(a) Lattice constants (*a*, *b*, *c*) and (b) volume with relative volume change estimated by *r*_vol_ = (*V*_*x*_ − *V*_0_)/*V*_0_ × 100(%), where *V*_0_ is the volume of the initial compound with *x* = 0, for the supercells (8 formula units, 48 atoms) of the Na_1+*x*_FeCl_4_ compound as the inserted Na content *x* varies from 0 to 1 in increments of 0.125, calculated using the optB88 XC functional with the Hubbard parameter *U*_eff_ = 2.0 eV. Dashed lines denote the unstable phases determined by analysis of the convex hull.

Using the same computational parameters, we then performed structural optimizations for the supercells of the intermediate phases Na_1+*x*_FeCl_4_, formed by inserting additional Na atoms into the NFC supercell with 8 fu (48 atoms), to simulate the discharge process in SIBs. Since 8 Na atoms can be inserted into the supercell on the whole, the Na content *x* varies from 0 to 1 in increments of 0.125, resulting in the formation of eight different intermediate phases. For each composition, there are several configurations, as the numbers can be calculated using the combination formula *C*_8_^*n*^ with *n* = 1–8. By considering the crystallographic symmetry, that is, taking the operation of all the point-group elements, the numbers of configurations were reduced to be 1, 6, 7, 13, 7, 6 and 1. For these configurations, we performed structural optimizations and recorded their total energies (see Table S3, SI), then selected the configuration with the lowest total energy (see Fig. S2 for optimal structures, SI). Fig. 2(a) shows the variation of the supercell lattice constants of the intermediate phases Na_1+*x*_FeCl_4_ as the inserted Na content *x* is varied from 0 to 1. With the exception of the intermediate phases with *x* = 0.375, 0.5 and 0.625, which turned out to be unstable from the analysis of the convex hull as discussed below, the lattice constants show smooth variations with the Na content *x*. As the Na content *x* increases, the lattice constant *a* was found to firstly increase and then decrease with relatively small changes, while the lattice constants *b* and *c* were found to gradually increase and decrease with clearly large changes. From this observation, it can be said that the repulsion between Na^+^ and Fe^3+/2+^ cations is dominant in the *b* direction, while the attraction between the cations and Cl^−^ anions is dominant in the *c* direction.

The volume change of the supercells was found to be up-and-down, as shown in Fig. 2(b). It should be noted that the highest volume observed at *x* = 0.25 is associated with the enhancement of repulsion between the cations, whereas the volumes lower than *V*_0_ for *x* = 0.75 are attributed to the enhancement of Na–Cl attraction. To estimate the cycling stability during the charge/discharge process, we evaluated the relative volume change rate by *r*_vol_ = (*V*_*x*_ − *V*_0_)/*V*_0_ × 100 (%). As shown in Fig. 2(b), all the values of *r*_vol_ are below 5%, indicating that the NFC compound exhibits superior cycling stability for SIB applications.

### 3.2 Energetics and electrode potential

**Fig. 3:**
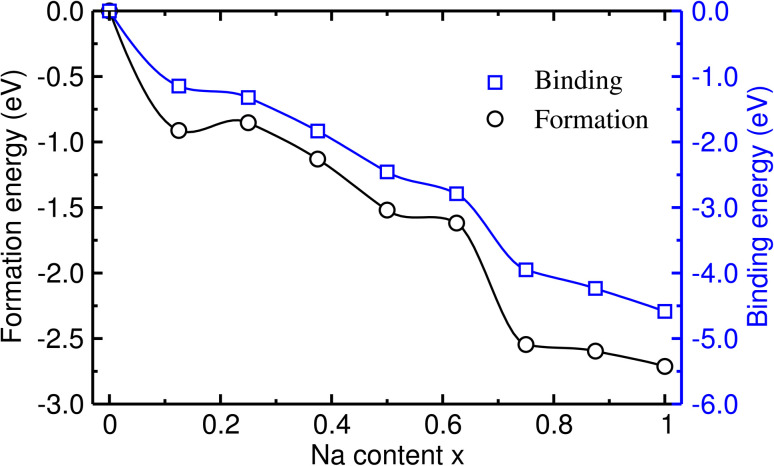
Calculated formation and binding energies in Na_1+*x*_FeCl_4_ as functions of Na content *x*.

Next, we estimated the formability of the intermediate phases Na_1+*x*_FeCl_4_ by calculating their formation energies (*E*_f_) from the NFC host and Na metal as follows:

2 *E*_f_ = *E*_Na_1+*x*_FeCl_4__ − (*E*_NaFeCl_4__ + *xE*_Na_bulk__),

where *E*_Na_bulk__ is the total energy per atom of bulk Na with the bcc (body-centered cubic) crystalline structure. A negative value of *E*_f_ indicates that the intermediate phase can be formed spontaneously, while a positive value indicates that the formation is an endothermic reaction. Fig. 3 shows the calculated formation energy as a function of Na content *x* ranging from 0 to 1. The formation energies were found to be negative for all the intermediate compounds, indicating their exothermic formation from the NFC host and Na metal. Moreover, one can find that the magnitude of *E*_f_ increases as the Na content *x* increases from 0 to 1, meaning that the formability of the intermediate compounds is enhanced with the increase of Na content.

To estimate the binding strength between the inserted Na atoms and the NFC host compound, we also calculated the binding energy *E*_b_ using the following equation:

3 *E*_b_ = *E*_Na_1+*x*_FeCl_4__ − (*E*_NaFeCl_4__ + *xE*_Na_atom__)

where *E*_Na_atom__ is the total energy of an isolated Na atom computed using the large cubic supercell (*a* = 15 Å) and only the Γ point of the BZ. As shown in Fig. 3, the calculated binding energies are negative for all the intermediate compounds, indicating attractive binding between the inserted Na atoms and the host. The magnitude of *E*_b_ was found to gradually increase with the increase of Na content *x*, indicating an enhancement of the attractive binding strength similarly with the formation energy.

**Fig. 4:**
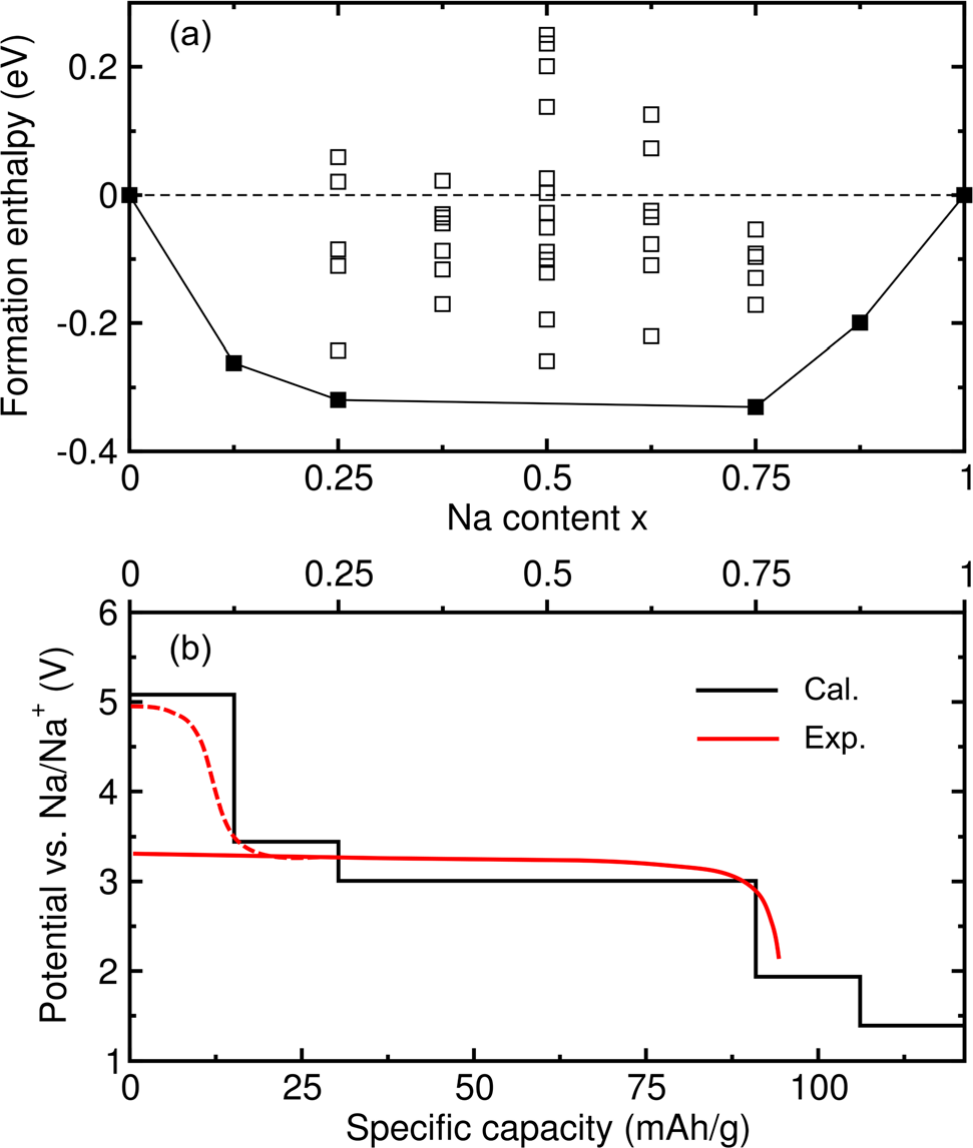
(a) Convex hull plot showing the formation enthalpies of the intermediate phases formed during Na-ion insertion as a function of Na content *x*. Black-coloured filled squares indicate stable phases whose formation enthalpies lie on the solid convex hull line, and the open squares denote the unstable phases. (b) Electrode potential step as a function of specific capacity, calculated from the formation enthalpies of the stable intermediate phases and compared with the experimental red-coloured curve.^14^

Then, we calculated the formation enthalpies of the intermediate phases with respect to the initial (NaFeCl_4_) and final (Na_2_FeCl_4_) compounds as follows:

4 Δ*H*_f_(*x*) = *E*_Na_1+*x*_FeCl_4__ − [(1 − *x*)*E*_NaFeCl_4__ + *xE*_Na_2_FeCl_4__],

where the inserted Na content *x* is varied from 0 to 1 with increments of 0.125 in this work. The formation enthalpies calculated in this way were used to estimate the relative stability and probability of formation during the charge/discharge process, and will be used to compute the electrode potential as discussed below. Fig. 4(a) shows the calculated Δ*H*_f_ for all the compositions and all the possible configurations at each composition, from which the convex hull curve was plotted. In the figure, the black-coloured filled squares indicate the phases that have their formation enthalpies placed on the convex hull curve, and these are regarded as the stable phases being formed during the discharge process. It was found that the intermediate phases with *x* = 0.125, 0.25, 0.75 and 0.875 were the stable ones owing to their formation enthalpies laid on the convex hull curve. However, the phases with *x* = 0.375, 0.5 and 0.625 were found to be unstable due to their formation enthalpies being above the convex hull, and therefore these phases could not be formed in the charge/discharge process.

The electrode potential is one of the most important quantities used to determine the performance of batteries, *i.e.*, the energy density of batteries. Using the convex hull approach, we can evaluate the electrode potential step for the identified stable intermediate phases. The step-like electrode potential *versus* the Na/Na^+^ counter-electrode was calculated using the DFT total energies of the neighbouring intermediate phases and bulk Na in the bcc structure as follows:^57^5
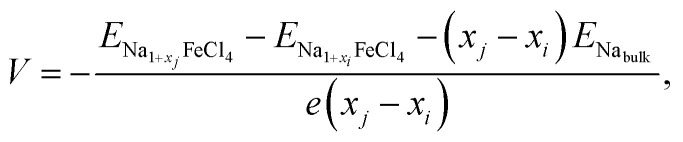
where 
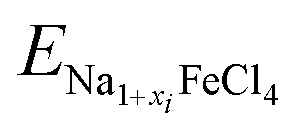
 is the total energy of the intermediate compound with a Na content of 1 + *x*_*i*_. Fig. 4(b) shows the calculated step-like electrode potential as a function of specific capacity, ranging from 0 to the maximum value of 121.25 mAh g^−1^, in comparison with the experimental curve.^14^ One can observe a relatively wide plateau region of electrode potential between *x* = 0.125 (15.16 mAh g^−1^) and *x* = 0.75 (90.94 mAh g^−1^), with an average electrode potential of 3.44 V, in good agreement with the experimental value of 3.45 V.^14^ From the calculation results, it can be concluded that the NFC compound can be used as a cathode material in ASSIBs due to its high electrode potential and wide plateau region.

### 3.3 Ionic conductivity

**Fig. 5:**
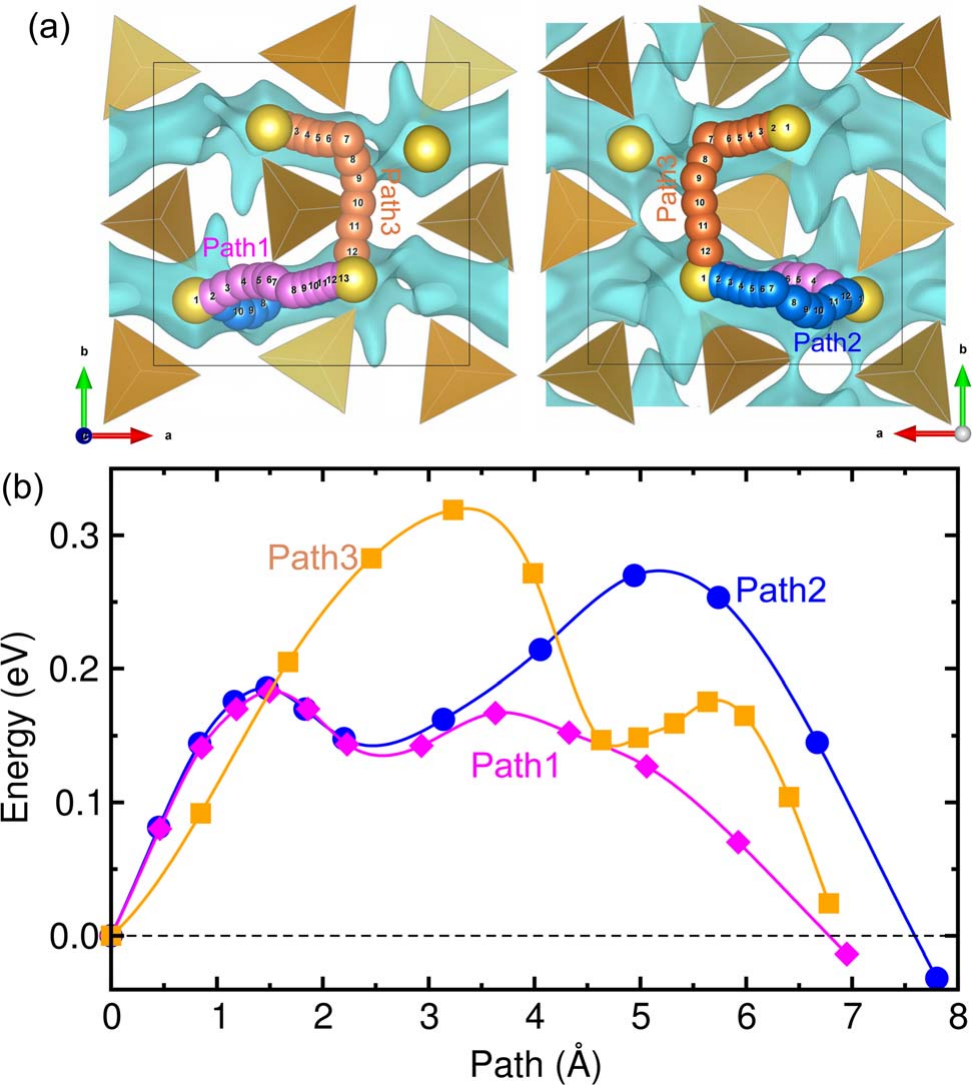
(a) Three different pathways for Na-ion migration identified by the blue-coloured isosurface of BVS and (b) the calculated energy profile for Na-ion migration along the three pathways.

The ionic conductivity is of great importance in determining the rate capability of alkali-ion batteries. In addition, the cycling life of the battery and the relative volume expansion rate are also influenced by ionic conductivity. As in most cathode materials of SIBs, Na-ion diffusion in NFC is suggested to occur *via* a vacancy-mediated mechanism in this work. As discussed above, we identified possible pathways for Na-ion migration by plotting the BVS isosurface, finding three different pathways, such as an almost linear pathway along the (101) direction (Path1), a Γ-type pathway along the (100) and (001) directions (Path2) and a Γ-type pathway along the (100) and (010) directions (Path3), as depicted in Fig. 5(a) (see also Fig. S3 and S4, SI). Note that these three pathways pass through the positions of the inserted Na atoms, and the channels of Path2 and Path3 appear to pass through the narrow middle part, as depicted by the isosurface of BVS, suggesting relatively higher activation energies compared with that of Path1. The activation energies for vacancy-mediated Na-ion migration along the three pathways were calculated by applying the CI-NEB method.^55^

Fig. 5(b) presents the computed energy profile for Na-ion diffusion. From CI-NEB calculations, the activation energies (*E*_a_) were determined to be 0.167, 0.270 and 0.272 eV along the three pathways, respectively. From the calculations, we can assert that Path1 is the fastest pathway for Na-ion diffusion due to its lowest *E*_a_ value of 0.167 eV, as expected from the BVS analysis. Nevertheless, the other two pathways are not ruled out since the activation energies are still relatively low compared with those of other conventional cathode materials of SIBs, such as layered oxide Na_0.44_MnO_2_ (∼0.37 eV)^58,59^ and polyanion compounds Na_*x*_Fe(SO_4_)_2_ (∼0.80 eV).^40,42,60^ From the calculated activation energies, we evaluated the diffusion coefficients using the equation *D* = *a*^2^*ν* exp(−*E*_a_/*k*_B_*T*), where *a* = 4 Å, *ν* = 10^11^ Hz and *k*_B_*T* = 0.026 eV at *T* = 300 K.^61^Table 1 lists the calculated diffusion coefficients for the three pathways in comparison with the experimental data.^14^ Along the Path1 channel with *E*_a_ = 0.167 eV, the *D* value was found to be as high as 2.60 × 10^−7^ cm^2^ s^−1^, whereas, along the Path2 and Path3 channels, the *D* values were 4.95 × 10^−9^ and 4.58 × 10^−9^ cm^2^ s^−1^, respectively, in good agreement with the experimental value of ∼5 × 10^−9^ cm^2^ s^−1^ at *T* = 333 K.^14^ The discrepancy is likely due to the fact that our DFT calculations were performed at 0 K, in contrast to the experiments conducted at a finite temperature, at which the crystalline order could be disturbed to some degree. From the calculation results, we maintain that NFC has a high Na ionic conductivity and thus supports a good rate capability of SIBs.

**Table 1:** Activation energy (*E*_a_) and Na-ion diffusion coefficient (*D*) in NaFeCl_4_ calculated (Cal.) in this work with the available experimental (Exp.) data.^14^

Path	*E*_a_ (eV)	*D* (×10^−8^ cm^2^ s^−1^)	
		Cal.	Exp.^14^
Path1	0.167	25.980	
Path2	0.270	0.495	0.5 (333 K)
Path3	0.272	0.458	0.001 (298 K)

### 3.4 Electronic and phonon properties

**Fig. 6:**
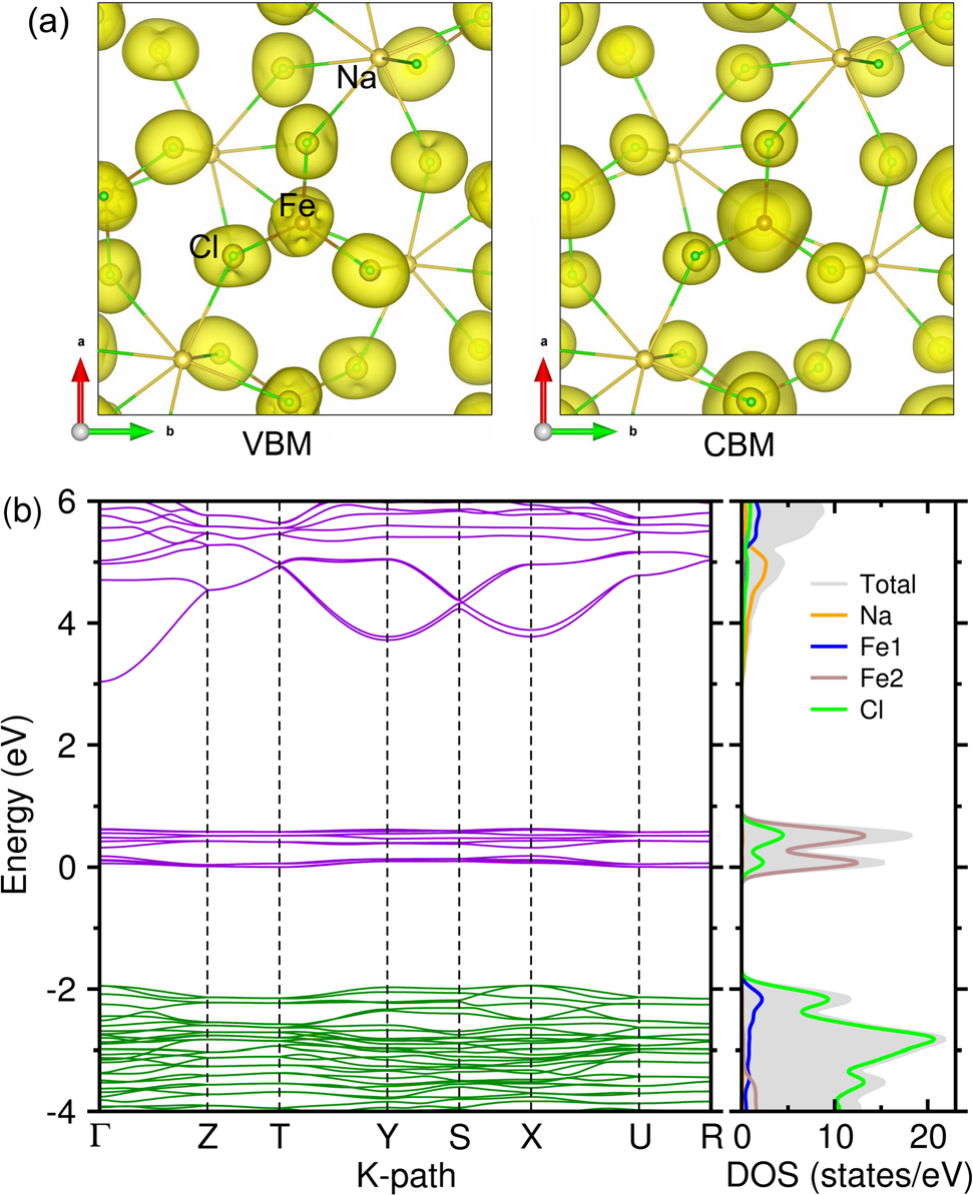
(a) Isosurface plot of squared wave functions corresponding to the VBM and CBM, (b) electronic band structure with atom-resolved density of states (DOS) in NaFeCl_4_, calculated using the optB88 XC functional and *U*_eff_ = 2.0 eV. Green(violet)-coloured lines present the valence (conduction) bands.

Electronic properties are also important for estimating the electron conduction during the charge/discharge process in electrode materials. Therefore, we calculated the electronic band structure and density of states (DOS) using the unit cell of NFC, the optB88 XC functional and the DFT+*U*_eff_ method with *U*_eff_ = 2.0 eV for the Fe 3d states. Fig. 6 shows the calculated band structure along the high-symmetry points of the BZ, together with the atom-resolved DOS and isosurface plots of the squared wave functions corresponding to the VBM and CBM. The band structure reveals an indirect band gap of ∼2 eV and very flat valence and conduction bands around the Fermi level, indicating that NFC is an insulating solid and that the charge carrier mobility is low. Therefore, it is recommended that an additive such as carbon black or graphene should be mixed with NFC during the preparation of SIB cathodes to enhance the electronic conductivity.

To clarify the contribution from each atom, we analyzed the atom-resolved DOS. It was found that the electronic states around the VBM were dominated by Cl 3p and Fe 3d spin-up electronic states, while those around the CBM were mostly attributed to Fe 3d spin-down states in hybridization with Cl 3p states (Fig. S6 and S7, SI). The Na 2p and 3s states were found to be away from the Fermi level (−23 and 5 eV from *E*_F_) (Fig. S5, SI). In addition, the Fe 4s states were found around −6 eV and the Fe 4p states were found to slightly contribute to the CBM. The Cl 3s states were found around −16 eV, indicating a negligible contribution to the VBM.

To obtain a meaningful understanding of electron transfer upon Na insertion into the host, we calculated the electronic charge density difference as follows:

6 Δ*ρ*(***r***)=*ρ*_Na_1.125_FeCl_4__(***r***) − [*ρ*_NaFeCl_4__(***r***) + 0.125*ρ*_Na_(***r***)],

where *ρ*_comp_(***r***) is the electronic density of the corresponding compound. During the calculation, the lattice sizes of the supercells were fixed to be the same as each other. It was found that the redistribution of electronic charge density occurred near Fe, Cl, and the inserted Na atoms (Fig. S8, SI). The electronic charge depletion was mostly found around the inserted Na atoms, while both charge depletion and accumulation were found around the nearby Fe and Cl atoms. This indicates that during the formation of NaFeCl_4_, the Na and Fe atoms offer 3s and 3d electrons, respectively, to become Na^+^ and Fe^3+^ cations, while the Cl atom receives an electron to become a Cl^−^ anion, and upon insertion of an Na atom, an electron moves from the inserted Na atom to the Fe^3+^ cation to become Na^+^ and Fe^2+^ cations. From this interpretation, it can be clarified that the Fe atoms act as a redox centre by changing oxidation states as Fe^3+^/Fe^2+^ on removal/insertion of Na atoms.

Finally, we estimated the thermodynamic stability of the NFC crystalline solid by computing the phonon dispersion curves and the atom-resolved phonon density of states (PDOS). Fig. 6(a) shows the phonon dispersion along the high-symmetry points of the phonon BZ, calculated at 0 K. The results showed the presence of anharmonic soft modes with imaginary phonon energies (red-coloured lines), that were relatively strong around the X point and weak at the Γ point, indicating that NFC in a monoclinic phase is thermodynamically unstable at 0 K. The soft modes were attributed to Cl atoms as can be seen in the PDOS. To check whether NFC is stable at finite temperatures, we performed phonon calculations at finite temperatures of 100, 200 and 300 K using the SCP approach.^54^

**Fig. 7:**
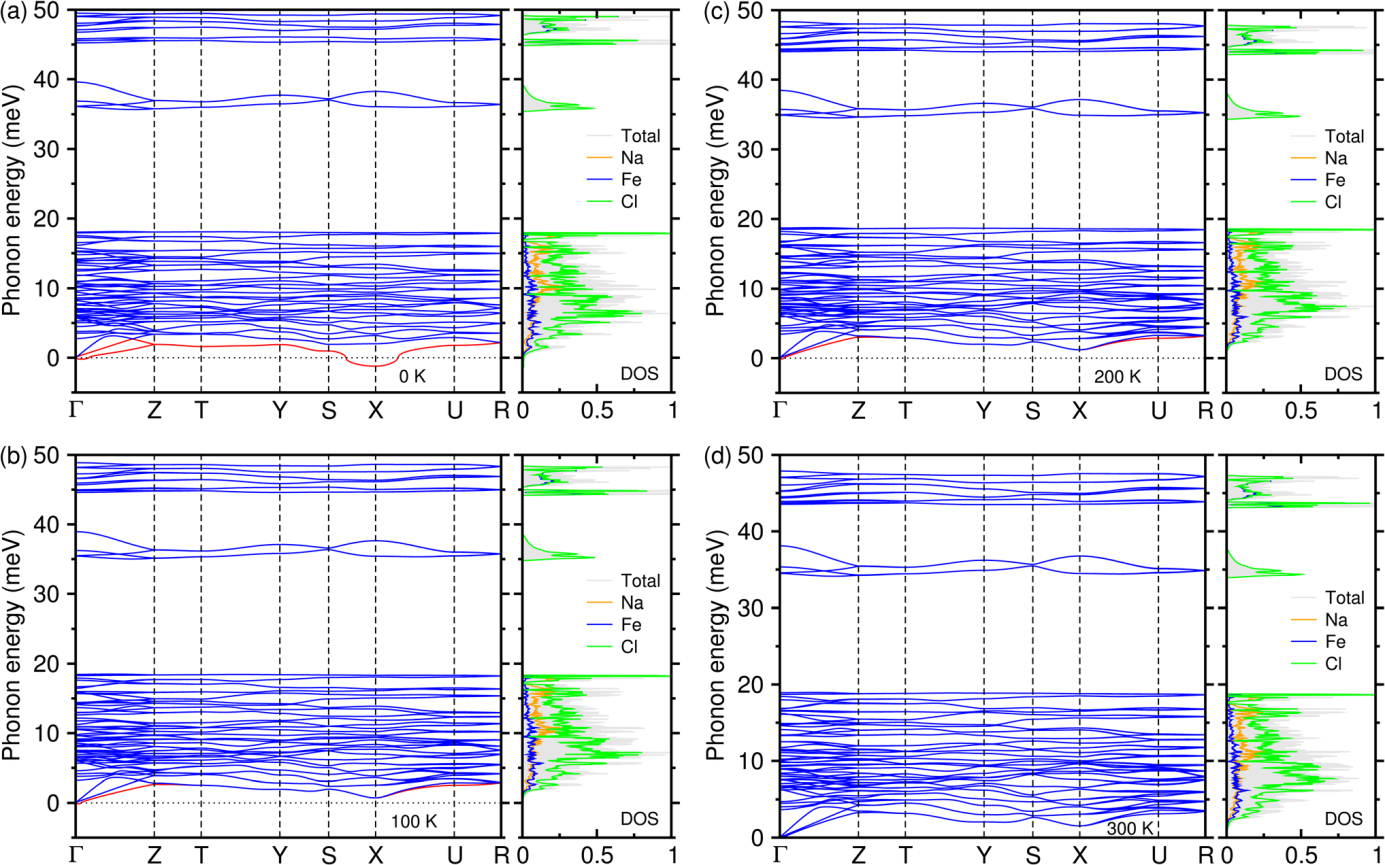
Phonon dispersion curves (left panel) and atom-resolved phonon density of states (right panel) of NaFeCl_4_ in a monoclinic phase with the *P*2_1_2_1_2_1_ space group at (a) 0 K, (b) 100 K, (c) 200 K and (d) 300 K. The red-coloured lines found at 0, 100 and 200 K indicate anharmonic soft modes with imaginary phonon energies.

At *T* = 100 and 200 K, the degenerate soft modes at the X point became normal modes with positive phonon energies, but the non-degenerate soft modes at the Γ point remained with small imaginary phonon energies of 0.123*i* and 0.119*i*, respectively, as shown in Fig. 7(b) and (c). This suggests the potential implications of the low-temperature instabilities for materials synthesis and battery operation. However, these soft modes can be ignored due to them having magnitudes smaller than 0.2, indicating that NFC in the monoclinic phase can be stable at over 100 K. It is noted that the ternary compound NaFeCl_4_ is relatively stable on the Na–Fe–Cl ternary phase diagram from the Materials Project^62^ (see Table S4 and Fig. S9, SI). The remaining non-degenerate soft modes at the Γ point disappeared completely at 300 K, as shown in Fig. 7(d). This indicates that the host NFC crystalline solid in the monoclinic phase becomes thermodynamically stable at room temperature and thus can be used safely as a cathode in SIBs. From the calculated PDOS, we found that the acoustic modes originated almost equally from all the atomic species, the middle-lying optical modes were dominated by Cl and Na atoms, and the high-lying optical modes were governed by Cl and Fe atoms.

## 4 Conclusions

In conclusion, we have investigated the electrochemical properties of the crystalline NaFeCl_4_ solid, which can be used as a cathode material of ASSIBs, using first-principles calculations. By using the BVS analysis and considering the crystalline symmetry, we predicted the additional positions for inserting Na atoms into a doubled supercell, forming Na_1+*x*_FeCl_4_ (*x* = 0–1) as intermediate phases, and determined the optimized structure with the lowest energy among different configurations for each Na composition. Our calculations showed a smooth change in lattice constants for the stable intermediate phases with increasing Na content *x*, confirming the relative volume changes of less than 5% and thus the long cycling life of SIBs. Through the calculation of formation enthalpies and plotting of the convex hull plot for Na_1+*x*_FeCl_4_, we calculated the step-like electrode potential, finding a wide plateau region with an average voltage of 3.45 V and a maximum specific capacity of 121 mAh g^−1^. For the ionic conductivity, we identified three different plausible pathways for Na migration and determined the corresponding activation energies, demonstrating fast Na-ion diffusion with a low activation energy of less than 0.3 eV, and diffusion coefficients in good agreement with the experimental data. Finally, we calculated the electronic band structures, finding that NFC is insulating with a band gap of 2 eV, and phonon dispersions at different temperatures, verifying that the NFC crystalline solid in the monoclinic phase is thermodynamically stable at room temperature. This work provides an atomistic insight into the electrochemical properties of NaFeCl_4_ and contributes to the design of advanced cathode materials for ASSIBs using iron-based chloride materials.

## Author contributions

Suk-Gyong Hwang performed the calculations and drafted the first manuscript. Tae-Il Ri, Ryo-Gyong Choe and Chung-Hyok Rim assisted with the post-processing of calculation results, and contributed to useful discussions. Chol-Jun Yu developed the original project and supervised the work. All authors reviewed the manuscript.

## Conflicts of interest

There are no conflicts to declare.

## Supplementary Material

RA-015-D5RA06124E-s001

## Data Availability

The data supporting this article have been included as part of the supplementary information (SI). Supplementary information: details on input files for calculations, tables for lattice parameters and atomic coordinates, and figures for structures of intermediate phases, BVS plot, atomic orbital-resolved DOS and charge density difference. See DOI: https://doi.org/10.1039/d5ra06124e.
