# Supplementary material for: First-principles study of the electrochemical properties of NaFeCl4 for cathode applications in sodium-ion batteries
Source: RSC Adv. 2025 Dec 19;15(59):51018–26. doi: 10.1039/d5ra06124e (PMC12716243; doi:10.1039/d5ra06124e)
Supplement: RA-015-D5RA06124E-s001 [file RA-015-D5RA06124E-s001.pdf]

Supplementary Information (SI) for RSC Advances.
This journal is © The Royal Society of Chemistry 2025

Electronic Supplementary Information – First-principles study on electrochemical properties of $NaFeCl_4$ for cathode application of sodium-ion battery

Suk-Gyong Hwang, Tae-Il Ri, Ryo-Gyong Choe, Chung-Hyok Rim and Chol-Jun Yu*

Computational Materials Design, Faculty of Materials Science, Kim Il Sung University, Taesong District, Pyongyang, Democratic People's Republic of Korea

Input files

The input file for self-consistent field (SCF) calculation for electronic band structure of $NaFeCl_4$ unit cell with antiferromanetic (AFM) configuration using the pw.x code is provided below.

```
&CONTROL
  calculation  = 'scf',
  restart_mode = 'from_scratch',
  outdir       = 'ebanddos-spin',
  prefix       = 'ebanddos-spin',
  pseudo_dir   = '/home/pub/pseudo/upf_files/gbrv/',
/
&SYSTEM
  ibrav = 0, celldm(1) = 1.88973,
  ntyp = 4, nat = 24,
  ecutwfc = 50, ecutrho = 400,
  input_dft = 'optbk88',
  occupations = 'smearing', smearing = 'm-p', degauss = 0.02
  nspin = 2,
  starting_magnetization(1) = 1,
  starting_magnetization(2) =-1,
  starting_magnetization(3) = 0,
  starting_magnetization(4) = 0
/
&electrons
  electron_maxstep = 100,
  conv_thr         = 1.0d-10,
  mixing_mode      = 'local-TF',
  mixing_beta      = 0.4,
  mixing_fixed_ns  = 4
/

ATOMIC_SPECIES
  Fe1  55.847    fe_pbesol_v1.5.uspp.F.UPF
  Fe2  55.847    fe_pbesol_v1.5.uspp.F.UPF
  Na   22.990    na_pbesol_v1.5.uspp.F.UPF
  Cl   35.453    cl_pbesol_v1.4.uspp.F.UPF

HUBBARD (ortho-atomic)
  U Fe1-3d 2.0
  U Fe2-3d 2.0

CELL_PARAMETERS (alat = 1.88973)
```

*Corresponding author: Chol-Jun Yu, Email: cj.yu@ryongnamsan.edu.kp

```
  10.18390   0.00000   0.00000
   0.00000   9.78733   0.00000
   0.00000   0.00000   6.15765

ATOMIC_POSITIONS (crystal)
  Fe1   0.03379    0.48122    0.70657
  Fe1   0.46621    0.51878    0.20657
  Fe2   0.96621    0.98122    0.79343
  Fe2   0.53379    0.01878    0.29343
  Na    0.13412    0.21530    0.18139
  Na    0.36588    0.78470    0.68139
  Na    0.86588    0.71530    0.31861
  Na    0.63412    0.28470    0.81861
  Cl    0.03131    0.48539    0.05262
  Cl    0.46869    0.51461    0.55262
  Cl    0.96869    0.98539    0.44738
  Cl    0.53131    0.01461    0.94738
  Cl    0.14887    0.30913    0.61854
  Cl    0.35113    0.69087    0.11854
  Cl    0.85113    0.80913    0.88146
  Cl    0.64887    0.19087    0.38146
  Cl    0.34053    0.03033    0.42627
  Cl    0.15947    0.96967    0.92627
  Cl    0.65947    0.53033    0.07373
  Cl    0.84053    0.46967    0.57373
  Cl    0.38604    0.34323    0.04930
  Cl    0.11396    0.65677    0.54930
  Cl    0.61396    0.84323    0.45070
  Cl    0.88604    0.15677    0.95070

K_POINTS {automatic}
  4 4 6 0 0 0
```

Then, the input file for non-SCF calculation for band structure is given as follows,

```
&CONTROL
  calculation  = 'bands',
  restart_mode = 'from_scratch',
  outdir       = 'ebanddos-spin',
  prefix       = 'ebanddos-spin',
  pseudo_dir   = '/home/pub/pseudo/upf_files/gbrv/',
/
&SYSTEM
  ibrav = 0, celldm(1) = 1.889726,
  ntyp = 4, nat = 24,
  ecutwfc = 50, ecutrho = 400,
  input_dft = 'optbk88',
  occupations = 'smearing', smearing = 'm-p', degauss = 0.02
  nbnd = 200,
  nspin = 2,
  starting_magnetization(1) = 1,
  starting_magnetization(2) =-1,
  starting_magnetization(3) = 0,
  starting_magnetization(4) = 0
/
&electrons
  ...
/

ATOMIC_SPECIES
  ...

HUBBARD (ortho-atomic)
```

```
  ...

CELL_PARAMETERS (alat = 1.889726)
  ...

ATOMIC_POSITIONS (crystal)
  ...

K_POINTS crystal_b
8
 0.0  0.0  0.0 30
 0.0  0.0  0.5 20
-0.5  0.0  0.5 30
-0.5  0.0  0.0 20
-0.5  0.5  0.0 20
 0.0  0.5  0.0 30
 0.0  0.5  0.5 20
-0.5  0.5  0.5 1
```

where "..." means the same to the corresponding part of SCF calculation. The input file for DOS calculation is given as follows.

```
&CONTROL
  calculation  = 'nscf',
  restart_mode = 'from_scratch',
  outdir       = 'ebanddos-spin',
  prefix       = 'ebanddos-spin',
  pseudo_dir   = '/home/pub/pseudo/upf_files/gbrv/',
/
&SYSTEM
  ibrav = 0, celldm(1) = 1.889726,
  ntyp = 4, nat = 24,
  ecutwfc = 50, ecutrho = 400,
  input_dft = 'optbk88',
  occupations = 'tetrahedra',
  nspin = 2,
  starting_magnetization(1) = 1,
  starting_magnetization(2) =-1,
  starting_magnetization(3) = 0,
  starting_magnetization(4) = 0
/
&electrons
  ...
/

ATOMIC_SPECIES
  ...

HUBBARD (ortho-atomic)
  ...

CELL_PARAMETERS (alat = 1.889726)
  ...

ATOMIC_POSITIONS (crystal)
  ...

K_POINTS {automatic}
  6 6 8 0 0 0
```

The finite displacement method using the doubled supercell was used for phonon calculations as implemented in the ALAMODE code in conjunction with the QE program. The input file for generating the configuration files for quartic interatomic force constant matrix is given below.

```
&general
  PREFIX = nfc112
  MODE = suggest
  NAT = 48; NKD = 3
  KD = Fe Na Cl
  TOLERANCE = 1.0e-4
/

&interaction
  NORDER = 3  #1: harmonic, 2: cubic, 3: quartic
/

&cell
  1.889726 # factor in Bohr unit
  10.18390   0.00000   0.00000
   0.00000   9.78733   0.00000
   0.00000   0.00000  12.31530
/

&cutoff
  *-* none 5.7 4.1
/

&position
  1    0.03379    0.48122    0.35329
  1    0.46621    0.51878    0.10329
  1    0.96621    0.98122    0.39671
  1    0.53379    0.01878    0.14671
  1    0.03379    0.48122    0.85329
  1    0.46621    0.51878    0.60329
  1    0.96621    0.98122    0.89671
  1    0.53379    0.01878    0.64671
  2    0.13412    0.21530    0.09070
  2    0.36588    0.78470    0.34070
  2    0.86588    0.71530    0.15930
  2    0.63412    0.28470    0.40930
  2    0.13412    0.21530    0.59070
  2    0.36588    0.78470    0.84070
  2    0.86588    0.71530    0.65930
  2    0.63412    0.28470    0.90930
  3    0.03131    0.48539    0.52631
  3    0.14887    0.30913    0.30927
  3    0.34053    0.03034    0.21313
  3    0.38604    0.34323    0.02465
  3    0.46869    0.51461    0.27631
  3    0.35113    0.69087    0.05927
  3    0.15947    0.96967    0.46313
  3    0.11396    0.65677    0.27465
  3    0.96869    0.98539    0.22369
  3    0.85113    0.80913    0.44073
  3    0.65947    0.53034    0.03687
  3    0.61396    0.84323    0.22535
  3    0.53131    0.01461    0.97369
  3    0.64887    0.19087    0.19073
  3    0.84053    0.46967    0.28687
  3    0.88604    0.15677    0.47535
  3    0.03131    0.48539    0.02631
  3    0.14887    0.30913    0.80927
  3    0.34053    0.03034    0.71313
  3    0.38604    0.34323    0.52465
  3    0.46869    0.51461    0.77631
  3    0.35113    0.69087    0.55927
```

```
  3    0.15947    0.96967    0.96313
  3    0.11396    0.65677    0.77465
  3    0.96869    0.98539    0.72369
  3    0.85113    0.80913    0.94073
  3    0.65947    0.53034    0.53687
  3    0.61396    0.84323    0.72535
  3    0.53131    0.01461    0.47369
  3    0.64887    0.19087    0.69073
  3    0.84053    0.46967    0.78687
  3    0.88604    0.15677    0.97535
```

The input file for optimizing the quartic force constant matrix is given below.

```
&general
  PREFIX = nfc112-harm-anharm4
  MODE = optimize
  NAT = 48; NKD = 3
  KD = Fe Na Cl
/

&fitting
  DFSET = DFSET-harm-anharm4
/

&interaction
  NORDER = 3  #1: harmonic, 2: cubic, 3: quartic
/

&cell
  ...
/

&cutoff
  *-* none 5.7 4.1
/

&position
  ...
```

The input file for calculating harmonic phonon band is given below.

```
&general
  PREFIX = nfc112-harm
  MODE   = phonons
  FCSXML = nfc112-harm.xml
  NKD = 3
  KD = Fe Na Cl
/

&cell
  1.889726 # factor in Bohr unit
  10.18390   0.00000   0.00000
   0.00000   9.78733   0.00000
   0.00000   0.00000   6.15765
/

&kpoint
  1  # KPMODE = 1: line mode
  G  0.0 0.0 0.0  Z  0.0 0.0 0.5  51
  Z  0.0 0.0 0.5  T -0.5 0.0 0.5  51
  T -0.5 0.0 0.5  Y -0.5 0.0 0.0  51
  Y -0.5 0.0 0.0  S -0.5 0.5 0.0  51
  S -0.5 0.5 0.0  X  0.0 0.5 0.0  51
  X  0.0 0.5 0.0  U  0.0 0.5 0.5  51
```

```
  U  0.0 0.5 0.5  R -0.5 0.5 0.5  51
/
```

The input file for calculation harmonic phonon DOS is given below.

```
&general
  PREFIX = nfc112-harm
  MODE   = phonons
  FCSXML = nfc112-harm.xml
    emin = -100
    emax = 500
    delta_e = 0.1
  NKD    = 3
  KD     = Fe Na Cl
/

&cell
  ...
/

&kpoint
  2
  10 10 10
/

&analysis
  PDOS = 1
/
```

The input file for phonon band structures at finite temperature using self-consistent phonon method is given below.

```
&general
  PREFIX = nfc112_scph
  MODE   = SCPH
  NKD    = 3
  KD     = Fe Na Cl
  MASS   = 55.845 22.99 35.5
  FCSXML = nfc112-harm-anharm4.xml
  TMIN = 100; TMAX = 500; DT = 100
/

&scph
 KMESH_SCPH        = 2 2 1
 KMESH_INTERPOLATE = 2 2 1
 SELF_OFFDIAG = 0
 RESTART_SCPH = 0
 MIXALPHA = 0.1
 MAXITER = 500
 TOL_SCPH = 1.0e-10
/

&cell
  ...
/

&kpoint
  ...
/
```

Tables

Table S1. The lattice constants (a, b, c) and relative errors, volumes and mass densities of $NaFeCl_4$ calculated using the unit cell, various exchange-correlation (XC) functionals and Habbard U parameters.

Method	a (Å)	Error (%)	b (Å)	Error (%)	c (Å)	Error (%)	Volume (Å^3)	ρ (g/cm^3)
PBEsol, no$U$	10.0967	-2.01	9.6566	-2.26	6.0418	-3.10	589.0739	2.49
PBEsol, PBE-PP, no$U$	10.1001	-1.98	9.6608	-2.22	6.0413	-3.11	589.4757	2.49
PBE, no$U$	11.0130	6.88	10.6178	7.47	6.0822	-2.45	711.2138	2.06
PBE, PBEsol-PP, no$U$	10.8572	5.37	11.2203	13.57	6.0817	-2.46	740.8734	1.98
PBE, U=4.5	10.7998	4.81	10.4204	5.47	6.1974	-0.60	697.4449	2.10
optB88, PBEsol-PP, no$U$	10.2267	-0.75	9.8335	-0.47	6.1034	-2.11	613.7907	2.39
optB88, PBEsol-PP, U=2	10.1839	-1.17	9.7873	-0.94	6.1576	-1.24	613.7526	2.39
optB88, PBEsol-PP, U=3	10.6636	3.49	6.8322	-30.85	7.5681	21.38	551.3815	2.66
optB86, PBE-PP, no$U$	10.2247	-0.77	9.8300	-0.51	6.1041	-2.10	613.5085	2.39
Exp.	10.304		9.880		6.235		634.7449	2.31

Table S2. Crystallographic atomic coordinates in $NaFeCl_4$ unit cell in orthorhombic phase with a space group $P2_12_12_1$, determined by using the optB88 XC functional.

Atom	Exp.[a]			Cal.		
	a	b	c	a	b	c
Fe	0.0382	0.4886	0.2127	0.033794	0.481225	0.206571
Na	0.1187	0.2138	0.6970	0.134123	0.215297	0.681390
Cl	0.0339	0.4924	0.5622	0.031315	0.485393	0.552624
Cl	0.1504	0.3118	0.1131	0.148867	0.309134	0.118537
Cl	0.3413	0.0179	0.9238	0.340529	0.030335	0.926270
Cl	0.3718	0.3274	0.5732	0.386038	0.343229	0.549304

[a]Experimental data is from J. Phys. Chem. 69 (1965) 239–244.

Table S3. Total energies of supercells with 8 formula units (48 atoms) for each configuration of $Na_{1+x}FeCl_4$ with x = 0.0, 0.125, 0.25, 0.375, 0.5, 0.625, 0.75, 0.875 and 1.0 (units: Ry).

No.	Na0	Na1	Na2	Na3	Na4	Na5	Na6	Na7	Na8
	0.0	0.125	0.25	0.375	0.5	0.625	0.75	0.875	1.0
1	-3834.349555	-3930.076319	-4025.682801	-4121.167806	-4216.792572	-4312.342610	-4407.980000	-4503.475527	-4598.930981
2			-4025.637671	-4121.135894	-4216.754652	-4312.277450	-4407.886489		
3			-4025.559895	-4121.118782	-4216.711484	-4312.257970	-4407.861793		
4			-4025.544815	-4121.093566	-4216.699692	-4312.233138	-4407.842369		
5			-4025.482623	-4121.088182	-4216.692436	-4312.227378	-4407.839449		
6			-4025.459975	-4121.085094	-4216.670100	-4312.169922	-4407.817193		
7				-4121.054414	-4216.656548	-4312.139106			
8					-4216.638252				
9					-4216.624812				
10					-4216.559604				
11					-4216.522180				
12					-4216.501332				
13					-4216.493396				

Table S4. Formation energy per atom (E_f) and deviation above convex hull (ΔE_h) for materials included in the Na–Fe–Cl ternary phase diagram with MP (Materials Project) identifier.

Material	MP identifier[a]	E_f (eV/atom)	ΔE_h (eV/atom)
Na	mp-10172-R2SCAN	0.0	
Na	mp-1079952-GGA	0.086	
Fe	mp-13-R2SCAN	0.0	
Fe	mp-150-R2SCAN	0.11	0.11
Cl_2	mp-22848-R2SCAN	0.0	
Cl_2	mp-1008394-R2SCAN	0.0	0.0
Na_3Cl	mp-1064484-GGA	−0.883	0.136
Na_2Cl	mp-990084-GGA	−1.255	0.104
Na_3Cl_2	mp-1095060-GGA	−1.407	0.224
NaCl	mp-22851-GGA	−1.884	0.154
NaCl	mp-22862-R2SCAN	−2.038	
$NaCl_3$	mp-1189265-GGA	−0.939	0.080
$NaCl_7$	mp-1080771-R2SCAN	−0.222	0.287
$FeCl_4$	mp-1225050-R2SCAN	−0.647	0.147
$FeCl_3$	mp-583463-R2SCAN	−0.993	
$FeCl_2$	mp-23229-R2SCAN	−0.986	0.022
$FeCl_2$	mp-571096-R2SCAN	−1.008	
Na_6FeCl_3	mp-1212446-R2SCAN	−1.806	0.027
$NaFeCl_4$	mp-27514-R2SCAN	−1.363	

[a]A. Jain et al., APL Mater. 1 (2013) 011002.

Figures

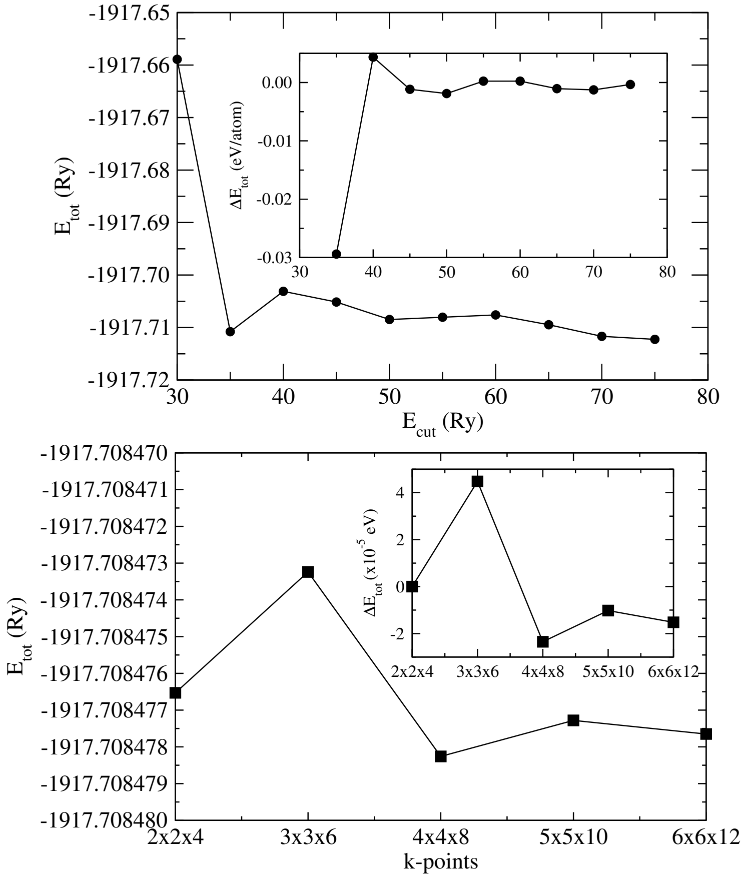

Fig. S1 Convergence test of total energy with respect to the cutoff energy (top) and k-point mesh (bottom).

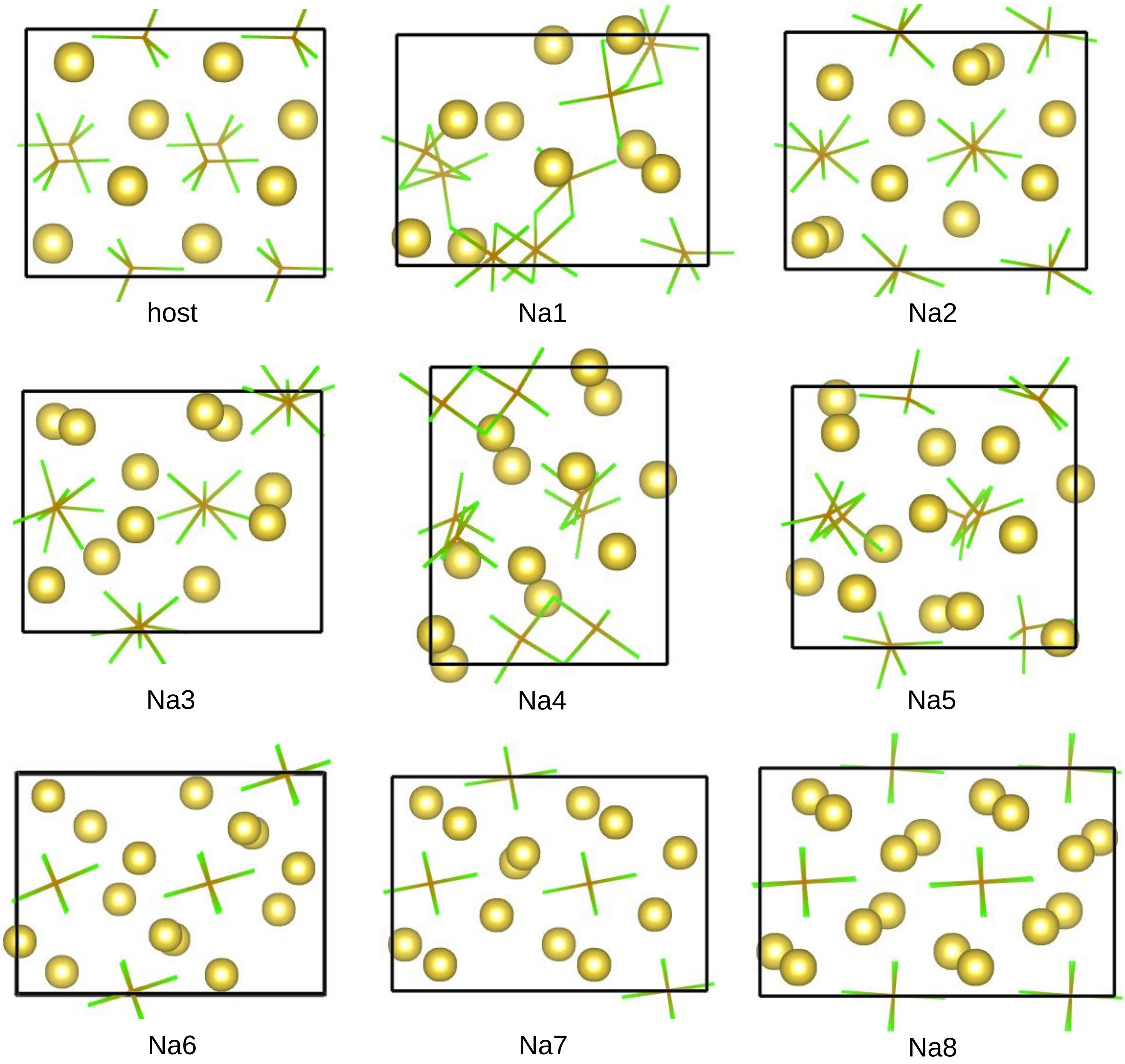

Fig. S2 Structures of $NaFeCl_4$ supercells as increasing the number of inserting Na atoms into the host supercell from 1 to 8.

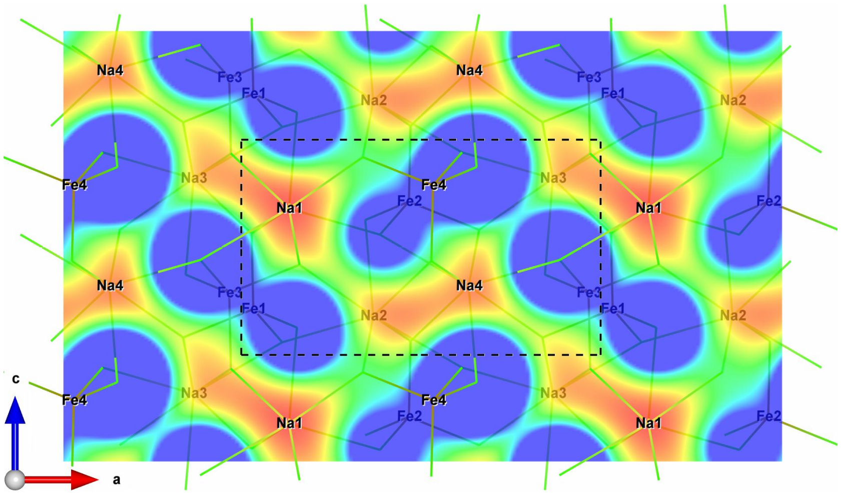

Fig. S3 Isosurface plot of BVS in $NaFeCl_4$ on the crystallographic $a - c$ plane. Red-coloured one end of dumbbell indicates the possible site for inserting Na atom.

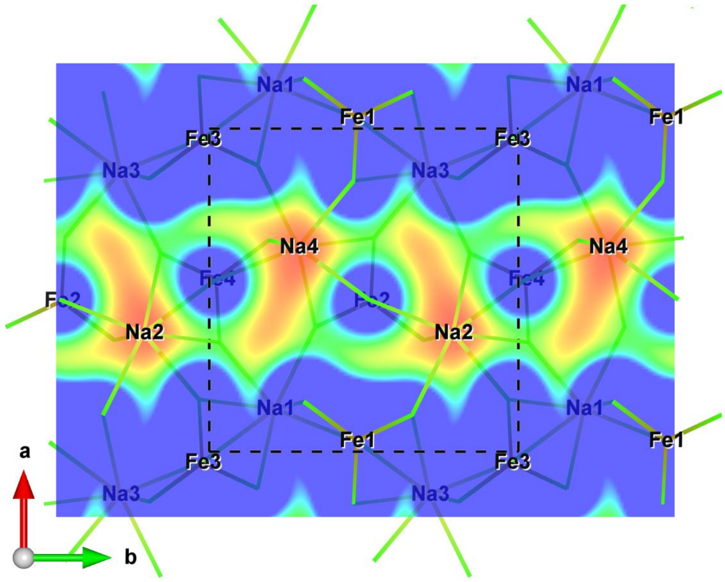

Fig. S4 Isosurface plot of BVS in $NaFeCl_4$ on the crystallographic $a - b$ plane.

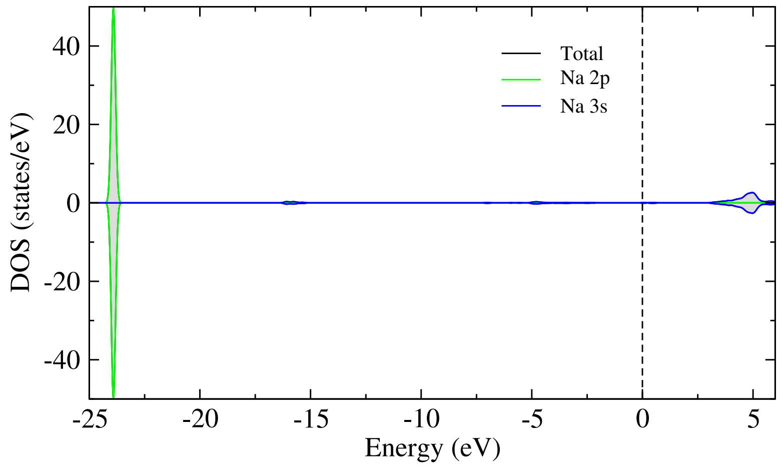

Fig. S5 Na atomic orbital-resolved partial density of states for Na 2p and 3s states in $NaFeCl_4$.

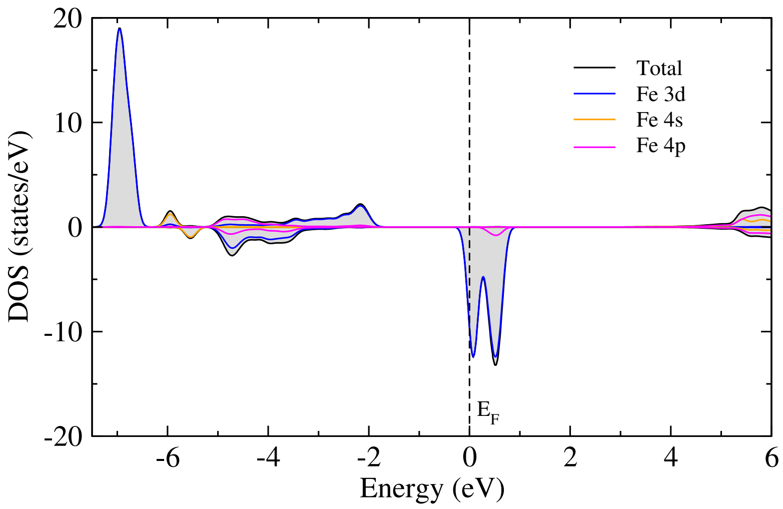

Fig. S6 Fe atomic orbital-resolved partial density of states for Fe 3d, 4s and 4p states in $NaFeCl_4$.

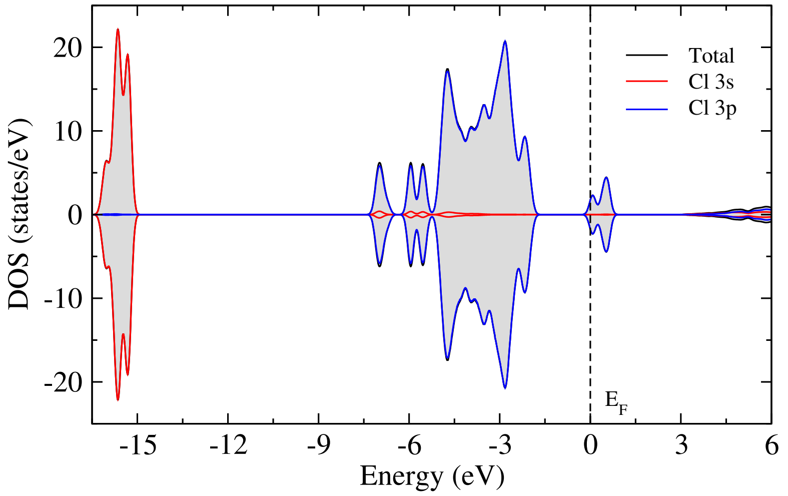

Fig. S7. Cl atomic orbital-resolved partial density of states for Cl 3s and 3p states in $NaFeCl_4$.

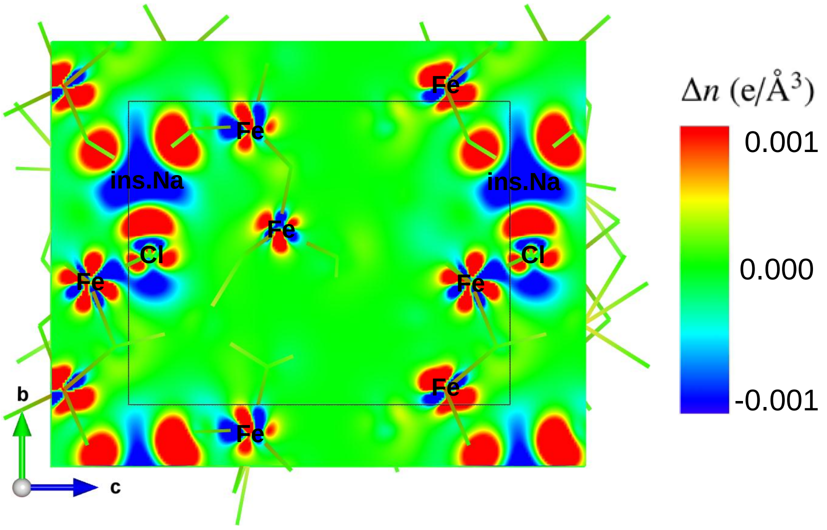

Fig. S8 Electronic density difference in $NaFeCl_4$ upon insertion of one Na atom. Positive (negative) values represent electron accumulation (depletion). Inserted Na atom is denoted as “ins. Na”.

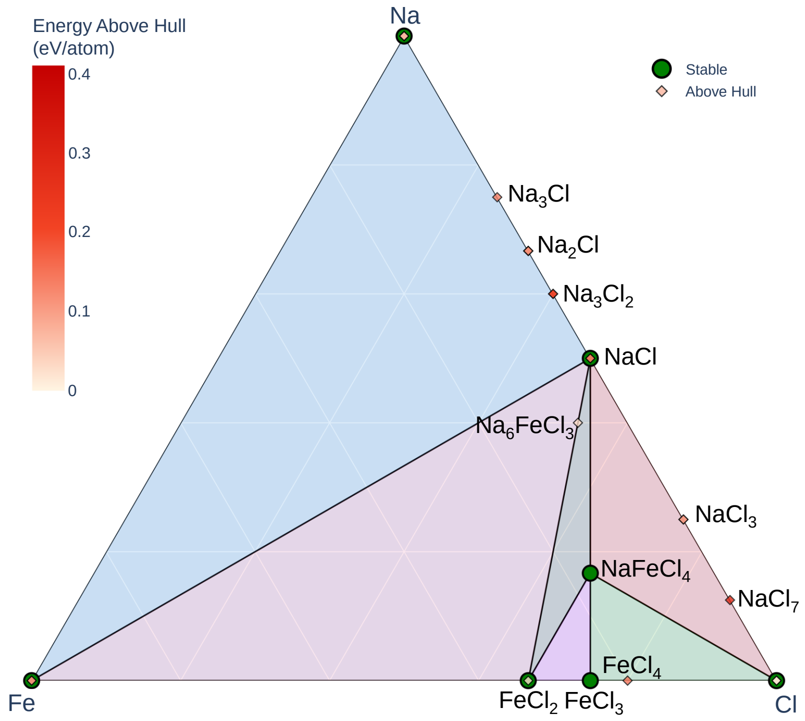

Fig. S9 Na–Fe–Cl ternary phase diagram extracted from Materials Project.
